# Current and Future Frontiers of Molecularly Defined Oligodendrogliomas

**DOI:** 10.3389/fonc.2022.934426

**Published:** 2022-07-25

**Authors:** Jordina Rincon-Torroella, Maureen Rakovec, Josh Materi, Divyaansh Raj, Tito Vivas-Buitrago, Abel Ferres, William Reyes Serpa, Kristin J. Redmond, Matthias Holdhoff, Chetan Bettegowda, José Juan González Sánchez

**Affiliations:** ^1^ Department of Neurosurgery, Johns Hopkins University School of Medicine, Baltimore, MD, United States; ^2^ Department of Neurosurgery, Hospital Clínic i Provincial, Barcelona, Spain; ^3^ Universidad de Santander (UDES), School of Medicine, Bucaramanga, Colombia; ^4^ Department of Radiation Oncology and Molecular Radiation Sciences, Johns Hopkins University School of Medicine, Baltimore, MD, United States; ^5^ Department of Oncology, Johns Hopkins University School of Medicine, Baltimore, MD, United States

**Keywords:** oligodendroglioma, diffuse glioma, 1p19q codeletion, EORTC, RTOG, POLCA, CODEL, NCCN

## Abstract

Oligodendrogliomas are a subtype of adult diffuse glioma characterized by their better responsiveness to systemic chemotherapy than other high-grade glial tumors. The World Health Organization (WHO) 2021 brain tumor classification highlighted defining molecular markers, including 1p19q codeletion and IDH mutations which have become key in diagnosing and treating oligodendrogliomas. The management for patients with oligodendrogliomas includes observation or surgical resection potentially followed by radiation and chemotherapy with PCV (Procarbazine, Lomustine, and Vincristine) or Temozolomide. However, most of the available research about oligodendrogliomas includes a mix of histologically and molecularly diagnosed tumors. Even data driving our current management guidelines are based on *post-hoc* subgroup analyses of the 1p19q codeleted population in landmark prospective trials. Therefore, the optimal treatment paradigm for molecularly defined oligodendrogliomas is incompletely understood. Many questions remain open, such as the optimal timing of radiation and chemotherapy, the response to different chemotherapeutic agents, or what genetic factors influence responsiveness to these agents. Ultimately, oligodendrogliomas are still incurable and new therapies, such as targeting IDH mutations, are necessary. In this opinion piece, we present relevant literature in the field, discuss current challenges, and propose some studies that we think are necessary to answer these critical questions.

## Introduction

Oligodendrogliomas are a subtype of adult diffuse glioma characterized by isocitrate dehydrogenase (IDH) mutation and the codeletion of the short arm of chromosome 1 (1p) and the long arm of chromosome 19 (19q) (1). They are rare primary brain tumors that present with variable outcomes and for which curative therapy does not exist. Oligodendrogliomas have evoked much interest given their favorable prognosis and better response to treatment compared to astrocytomas and glioblastomas, their more malignant counterparts. Historically, the diagnosis of oligodendrogliomas was purely histological, based on the characteristic “fried-egg” appearance of oligodendroglial cells, which was subject to considerable interobserver variation (2, 3). Exhaustive research led to discovering the 1p/19q codeletion, a molecular marker that has come to define oligodendroglioma (4–6). Hence, the diagnosis of oligodendroglioma became molecular instead of histological.

The WHO 2021 brain tumor classification reinforced this requirement and included 1p/19q codeletion and IDH mutation as defining traits of oligodendroglioma (1). Given that this change became official in relatively recent times, it is not surprising that pivotal prospective trials that guide current clinical decisions were based on the histological diagnosis of oligodendroglioma (7, 8). Retrospective research conducted before or even after that critical change studied a mix of histological and molecularly diagnosed oligodendrogliomas with the risk of including tumors that may not be classified as oligodendrogliomas now (9–15). There is a paucity of studies on purely molecularly defined oligodendrogliomas and those usually have a small number of patients or limited follow-up time (16–22).

Technological developments over the past decade allowed the implementation of genomic studies to our current standard of care for brain tumors, including complex next-generation sequencing (NGS) to decipher their genomic features. Consequently, patients may receive detailed results of NGS panels that describe several tumor-associated mutations. Although many advances have been made, we do not fully understand the clinical implications of those mutations and how a specific genomic signature affects an individual patient’s outcome. This is especially true for oligodendrogliomas, where follow-up lasting 10-20 years may be required to understand the impact on prognosis.

Here, we summarize the scientific basis of current management decisions, pose critical questions that remain unanswered, and highlight ongoing or future studies that can improve the management of patients with oligodendrogliomas.

## Oligodendroglioma: A Molecular Diagnosis

Oligodendrogliomas represent only 5%-10% of all glial tumors in population-based studies (23, 24). Although they typically occur in younger adults, they can appear at any age, have a higher incidence in men, and are rare in children (24, 25). More than 70% of oligodendrogliomas are WHO grade 2, and approximately 20% are WHO grade 3 (1, 24).

The diagnosis of oligodendrogliomas requires the presence of both 1p/19q codeletion and IDH mutation (1). Two landmark papers in 2015 were pivotal to adopting this change. The first was a population-based study of 1087 diffuse gliomas that analyzed the mutation status of 1p/19q, IDH1 and 2, and TERT promoter. Classifying grade 2 and 3 gliomas based upon those mutations stratified the tumors into five molecular subgroups that were independently associated with clinical outcomes. This included the “triple positive” group, which harbored 1p/19q codeletion, IDH, and TERT promoter mutations. Triple-positive gliomas were most strongly associated with the oligodendroglial histologic type and better overall survival (4). This strengthened the importance of harboring an IDH mutation in addition to a 1p19q codeletion to confer a better prognosis, which was already described by Cairncross et al. and Jiao et al. in prior studies (26, 27). The second landmark paper was a genome-wide study of 293 low-grade gliomas by the TCGA Research Network. The group identified three molecular subtypes of lower-grade gliomas using a wide array of genomic, methylation, and protein expression analyses. The IDH-methylated, the IDH-wildtype, and the 1p/19q codeleted subgroups were found to be three prognostically significant and non-overlapping subtypes. Those two molecular markers (IDH mutation and 1p/19q codeletion) became critical in the current diagnosis of gliomas (5). This study also confirmed previous reports by our team and others identifying CIC and FUBP1 as potential oligodendroglioma tumor suppressor genes lost on chromosomes 1p and 19q, respectively (27, 28). Other molecular mutations frequently reported in gliomas, including chromosome 9p deletion and subsequent CDKN2A gene loss, have also been postulated to be involved in oligodendroglioma pathogenesis and malignant progression (29). Markers that drive an aggressive phenotype specifically in anaplastic oligodendroglioma have also been reported, including the transcription factor TCF12 (29). The specific role of these and other molecular markers and their effect on survival remain unclear.

## Oligodendroglioma Management: Evidence And Controversies

Despite prior research, the optimal treatment paradigm for oligodendrogliomas is still in question (25). Management may start with surgery or observation. Many glioma studies have indicated that more extensive tumor resection with functional preservation is associated with prolonged survival (10–12, 30–33). Thus, although there is limited specific data on molecularly defined oligodendrogliomas, surgery for pathological diagnosis and maximal safe resection remains the favored initial therapeutic approach (31–34).

After surgical removal, upfront treatments for grade 2 oligodendrogliomas include observation (specifically in younger patients who underwent gross total resection [GTR]) or radiation with adjuvant chemotherapy. Grade 3 oligodendrogliomas are typically treated by surgical resection followed by radiation and chemotherapy, although observation may be an option in low-risk cases (35, 36). High-risk has been traditionally considered as being over 40 years of age or receiving less than GTR; however, this is controversial given the indolent biology of these tumors.

Current adjuvant treatment guidelines for oligodendrogliomas are based on two landmark clinical trials in anaplastic oligodendrogliomas and anaplastic oligoastrocytomas. These were the EORTC 26951 by the European Organization for Research and Treatment of Cancer and RTOG 9402 by the Radiation Therapy Oncology Group (7, 8). Both studies compared the role of Procarbazine, Lomustine - also known as CCNU, and Vincristine (PCV) in combination with radiotherapy with that of radiation therapy alone. However, the timing of radiotherapy was not evaluated. In addition, these trials were designed before discovering the prognostic implications of 1p/19q codeletion and IDH mutations. The 1p/19q codeletion was detected in 48% (126 of 263) of the cases in the RTOG and only 25% (80 of 316) in the EORTC trial (7, 8). In *post-hoc* analyses, both trials demonstrated that tumors with a 1p/19q codeletion benefitted from adding PCV to radiation therapy, markedly increasing the overall survival (OS) of patients with anaplastic oligodendroglioma. Specifically in RTOG 9402 the addition of PCV to RT improved OS from 7.3 to 14.7 years, and OS was not reached at the time of the EORTC 26951 data publication (7, 8). Studies attempting to identify the molecular determinants of survival from the RTOG 9402 were unsuccessful, largely due to a lack of sufficient samples (37).

The PCV regimen entails considerable side effects, including myelosuppression, hepatotoxicity, and neurotoxicity. Grade 3 or 4 hematologic toxicity was reported in more than 45% of the patients assigned to the experimental arm in the EORTC 26951 and RTOG 9402 trials (8, 38). Both studies were launched before the introduction of the oral alkylating agent temozolomide (TMZ) into neuro-oncology practice. TMZ has a more favorable toxicity profile than PCV and neuro-oncologists had become familiar with it in treating high-grade astrocytomas. With the RTOG 9402 and EORTC 26951 data pending, many neuro-oncologists started treating patients with the same regimen used for glioblastoma. TMZ resulted in considerable response rates and promising survival when used as “salvage” chemotherapy in oligodendroglioma relapse after the failure of PCV (39). However, retrospective studies of newly diagnosed oligodendrogliomas treated with TMZ alone revealed very variable outcomes with controversial conclusions (40, 41). The striking results of RTOG 9402 and EORTC 26951, two independently conducted, randomized studies with PCV, challenged the use of radiation and TMZ for oligodendrogliomas, as a comparable level of evidence did not exist for this regimen (25, 42, 43).

An ongoing international phase III clinical trial (CODEL, NCT00887146) was designed to resolve this mystery (44). The CODEL trial compares the efficacy of concomitant radiotherapy with TMZ followed by adjuvant TMZ versus radiotherapy with adjuvant PCV. It is well known that radiotherapy provides an improved progression-free survival for oligodendroglioma patients. In fact, the CODEL trial was initially designed to compare TMZ alone, radiotherapy alone, and radiotherapy with concomitant and adjuvant TMZ. Because the TMZ-alone patients experienced significantly shorter progression-free survival when compared to the patients in the radiotherapy arms, CODEL was redesigned to its current paradigm (44). Radiotherapy has shown promising efficacy with oligodendrogliomas, but there is significant concern for its long-term neurocognitive effects, and the timing of radiotherapy is questioned (16, 25). An ongoing clinical trial (POLCA, NCT02444000) investigates the difference between upfront radiotherapy with PCV versus upfront PCV with deferred radiotherapy. Another active phase III clinical trial (NCT00978458) conducted by the Eastern Cooperative Oncology Group and the National Cancer Institute (NCI) is evaluating whether the addition of TMZ to adjuvant radiation therapy improves survival in patients with low grade glioma, including oligodendroglioma (45). These studies may provide some answers to those critical questions, but the final results will not be available for years. In fact, the expected completion time for primary outcome data collection since the initiation of the CODEL and POLCA trials is 16 and 9 years, respectively (46, 47).

In light of those challenges, both the European Association of Neuro-Oncology (EANO) and the joint American Society of Clinical Oncology (ASCO) and Society for Neuro-Oncology (SNO) recognized the need for clarification by publishing recent evidence-based guidelines on the management of diffuse gliomas, including oligodendrogliomas (35, 36). There have also been changes in the National Comprehensive Cancer Network (NCCN) guidelines for oligodendrogliomas (48). A comparison of those guidelines is presented in **Table 1**.

**Table 1:** Societal guidelines for oligodendroglioma management.

Guidelines	American Society of Clinical Oncology and Society for Neuro-Oncology		European Association of Neuro-Oncology		NCCN Guidelines® for Central Nervous System Cancers	
WHO Grade	2	3	2	3	2	3
Molecular diagnosis	IDH1 or IDH2 mutation, 1p19q codeletion					
Surgical therapy	Maximal safe resection					
When to “wait and see”	Defer RT-CT *if*: Absent symptomatic or radiological progression Positive prognostic factors (e.g., complete resection younger age) or concerns about toxicity	NA	Defer RT-CT *if*: GTR, incomplete, resection, <40 y.o., and absent neuro deficits beyond symptomatic epilepsy	Defer RT-CT *if*: GTR, <40 y.o., no neurological deficits, and without homozygous *CDKN2A/B* deletion	Defer RT-CT *if*: Low-risk patients (e.g., GTR and ≤40 y.o) High-risk patients (e.g., >40 y.o. or STR or open/stereotactic biopsy) that are neurologically asymptomatic or stable	NA
Adjuvant Therapy	RT followed by CT					
Radiation therapy	54 Gy in 30 fractions over 6 wk	59.4 Gy in 33 fractions at 5 fractions per wk	50–54 Gy in 1.8–2 Gy fractions	54–60 Gy in 1.8–2 Gy fractions	45-54 Gy	59.4 Gy in 1.8 Gy fractions for 28 fractions followed by a 5-fraction boost of 1.8 Gy/fraction
Chemotherapy	RT followed by PCV: procarbazine 60 mg/m^2^ PO QD d 8 – 21, lomustine 110mg/m^2^ PO QD on d 1, vincristine 1.4 mg/m^2^ IV QD d 8 and 29 in 8 wk cycle for a total of six cycles C/f PCV toxicity, adjuvant TMZ 150-200 mg/m^2^ PO QD d 1-5, every 4 wk for a maximum of 12 mo		RT followed by PCV (PCV alone remains investigational, may reduce the risk of late cognitive deficits)	RT followed by PCV (PCV alone remains investigational, may reduce the risk of late cognitive deficits)	Consider clinical trial for those eligible RT followed by PCV RT with or without concurrent TMZ followed by adjuvant TMZ PCV or TMZ alone in rare circumstances	Consider clinical trial for those eligible RT with neoadjuvant or adjuvant PCV RT with or without concurrent TMZ followed by adjuvant TMZ
Surveillance	No recommendation		Neurological exam and imaging every 3-6 mo		MRI every 3-6 mo for 5 y then every 6-12 mo or as clinically indicated	MRI 2-8 wk after RT, then every 2-4 mo for 3 y, then every 3-6 mo indefinitely
Treatment at progression/recurrence	No recommendation		Based on response to first-line therapy, consider: - Repeat surgery - Re-irradiation - PCV - TMZ - Experimental therapy for WHO grade 3: bevacizumab^a^		Surgery if resectable Biopsy if unresectable Consider clinical trial for those eligible No prior RT, consider: - RT + adjuvant PCV - RT + adjuvant TMZ - RT + concurrent and adjuvant TMZ - CT alone - RT alone^b^ Prior RT, consider: - PCV - TMZ - Lomustine or carmustine - Platinum-based regimens - Reirradiation^c^ ± CT - Palliative/supportive care - Observation if low-risk MRI every 2-3 mo	Surgery if resectable Consider clinical trial for those eligible Systemic CT ± RT - RT + adjuvant PCV - RT + adjuvant TMZ - RT + concurrent and adjuvant TMZ - Re-irradiation^d^ - TMZ - Lomustine or carmustine - PCV - Platinum-based regimens Palliative/supportive care

C/f, concern for; CT, chemotherapy; GTR, gross total resection; d, days; Gy, gray; IDH, isocitrate dehydrogenase; mg/m^2^, milligram per square-meter; PCV, procarbazine/ lomustine/ vincristine; QD, once a day; mo, months; RT, radiation therapy; TMZ, temozolomide; WHO, World Health Organization; y, years; y.o., years old.

^a^ Used for symptom control.

^b^ RT alone is generally not the preferred treatment option except in select cases (e.g., poor performance status).

^c^ Consider if a new lesion outside the target of prior RT or recurrence is small and geometrically favorable.

^d^ Consider re-irradiation if long interval since prior RT and/or if there was a good response to prior RT.

Adapted from Weller M et al. EANO guidelines on the diagnosis and treatment of diffuse gliomas of adulthood. Nat Rev Clin Oncol. 2021;18(3):170–86., Mohile NA et al. Therapy for Diffuse Astrocytic and Oligodendroglial Tumors in Adults: ASCO-SNO Guideline. J Clin Oncol. 2022;40(4):403–26.NA, not applicable.

In summary, the current management for suspected oligodendroglioma consists of observation, surgery, radiation treatment, and chemotherapy. Observation is questionable given the strong evidence in favor of adjuvant therapy. Radiation treatment can be given after surgery and before chemotherapy, or chemotherapy can be given first, with radiation deferred to tumor progression. PCV is the chemotherapy of choice in the official guidelines, with TMZ reserved in those cases with PCV toxicity. In the past, both radiation and chemotherapy were usually delayed in the treatment of oligodendrogliomas. However, given the striking response to chemotherapy, the early use of adjuvant therapies has been favored in the past few years, especially in grade 3 oligodendrogliomas. For example, a 2019 study of the National Cancer Database showed that radiation followed by chemotherapy is the favored sequence of adjuvant therapy for grade 3 oligodendrogliomas in the US (13). Beyond current treatment, new therapeutic avenues are necessary and underway. Exciting work has been done targeting mutant IDH or related pathways. An important example is INDIGO (NCT04164901), an ongoing randomized phase III study of vorasidenib, a promising oral inhibitor of IDH1/2 mutations that has shown a 30.8% objective response rate in non-enhancing glioma patients (49). Other drugs that target molecular markers including abemaciclib, a CDK inhibitor selective for CDK4 and CDK6, are also being investigated for use in oligodendroglioma patients in ongoing clinical trials (NCT03969706) (50).

## Discussion

As described, many questions remain unanswered regarding the management of oligodendrogliomas, and a comprehensive understanding of current practices is not known. This was stressed in the oligodendroglioma workshop organized by the National Cancer Institute’s NCI-CONNECT in 2018 (25). However, in part due to the low incidence and high complexity of oligodendroglioma management, few of those questions have been clarified. In addition, available prospective data on the management of molecularly defined oligodendrogliomas are either indirect or from *post-hoc* subgroup analysis with potential risk of bias. More definitive answers may be provided by ongoing long-lasting multi-institutional clinical trials based on molecular criteria. In our opinion, intermediate answers are required to shed light on the current management of this type of tumor and standardize practices. First, it is paramount that any future oligodendroglioma study is based on the current molecular definition of oligodendroglioma, confirming 1p19q codeletion and IDH mutation. Second, we believe that surveying the oncological and neurosurgical societies would clarify if current treatment trends, especially adjuvant therapy utilization and chemotherapy regimen selection, differ between geographical regions given the current lack of universal and standardized worldwide guidelines. Although not definitive, an exhaustive study of the clinical features, management, genetic profile, and outcomes of purely molecularly defined oligodendrogliomas in a large retrospective cohort would potentially unveil characteristic features and provide updated management guidance based on current diagnostic standards. This can also help improve risk stratification to extend beyond age and extent of resection. Changes in medical practice are complex and require widespread dissemination of information. In the long run, a worldwide task force in charge of revising and implementing the CODEL and POLCA trial results will be essential to translate high-quality data into daily practices. Finally, oligodendrogliomas are still not curable, and new therapeutic avenues are imperative. Whether IDH inhibitors become integral to treating this disease remains to be evaluated. These and other important clinical trials are desperately needed to improve outcomes for patients with oligodendroglioma.

## Conclusion

Here, we have summarized the current advancements in the molecular characterization of oligodendrogliomas and reviewed adjuvant treatment modalities currently used in its treatment. The future directions in research we have outlined, including retrospective and clinical trials, have significant potential to further advance the management and prognosis of oligodendroglioma patients when effectively translated into clinical practice.

## Author Contributions

JR-T, CB, and JJGS devised the project, the main conceptual ideas, and the proof outline. JR-T, MR, JM, DR, TV-B, AF, WRS, KR, MH, CB, and JJGS contributed to the design and implementation of the research, the analysis of the results, and the writing of the manuscript. In addition, WRS, CB, and JJGS provided institutional support. All the authors approved and reviewed the submitted version of the manuscript.

## Funding

JR-T is an NINDS R25 training grant awardee (5R25NS065729).

## Conflict of Interest

CB is a consultant for Depuy-Synthes, Bionaut Labs and Galectin Therapeutics and a co-founder of OrisDx. KR: Research support from Elekta AB, Accuray, Canon; Data Safety Monitoring Board for BioMimetix; Travel expenses from Brainlab, Elekta AB, Accuray, Icotec, RSS; Honorarium for speaking engagement NCCN, Accuray.

The remaining authors declare that the research was conducted in the absence of any commercial or financial relationships that could be construed as a potential conflict of interest.

## Publisher’s Note

All claims expressed in this article are solely those of the authors and do not necessarily represent those of their affiliated organizations, or those of the publisher, the editors and the reviewers. Any product that may be evaluated in this article, or claim that may be made by its manufacturer, is not guaranteed or endorsed by the publisher.
